# Towards an explainable clinical decision support system for large-for-gestational-age births

**DOI:** 10.1371/journal.pone.0281821

**Published:** 2023-02-21

**Authors:** Yuhan Du, Anthony R. Rafferty, Fionnuala M. McAuliffe, John Mehegan, Catherine Mooney

**Affiliations:** 1 UCD Perinatal Research Centre, School of Computer Science, University College Dublin, Dublin, Ireland; 2 UCD Perinatal Research Centre, School of Medicine, University College Dublin, National Maternity Hospital, Dublin, Ireland; 3 UCD School of Public Health, Physiotherapy and Sports Science, University College Dublin, Dublin, Ireland; UNITED STATES

## Abstract

A myriad of maternal and neonatal complications can result from delivery of a large-for-gestational-age (LGA) infant. LGA birth rates have increased in many countries since the late 20^*th*^ century, partially due to a rise in maternal body mass index, which is associated with LGA risk. The objective of the current study was to develop LGA prediction models for women with overweight and obesity for the purpose of clinical decision support in a clinical setting. Maternal characteristics, serum biomarkers and fetal anatomy scan measurements for 465 pregnant women with overweight and obesity before and at approximately 21 weeks gestation were obtained from the PEARS (Pregnancy Exercise and Nutrition with smart phone application support) study data. Random forest, support vector machine, adaptive boosting and extreme gradient boosting algorithms were applied with synthetic minority over-sampling technique to develop probabilistic prediction models. Two models were developed for use in different settings: a clinical setting for white women (AUC-ROC of 0.75); and a clinical setting for women of all ethnicity and regions (AUC-ROC of 0.57). Maternal age, mid upper arm circumference, white cell count at the first antenatal visit, fetal biometry and gestational age at fetal anatomy scan were found to be important predictors of LGA. Pobal HP deprivation index and fetal biometry centiles, which are population-specific, are also important. Moreover, we explained our models with Local Interpretable Model-agnostic Explanations (LIME) to improve explainability, which was proven effective by case studies. Our explainable models can effectively predict the probability of an LGA birth for women with overweight and obesity, and are anticipated to be useful to support clinical decision-making and for the development of early pregnancy intervention strategies to reduce pregnancy complications related to LGA.

## Introduction

Large-for-gestational-age (LGA) newborns have a birth weight greater than the 90^*th*^ centile for their gestational age at delivery. LGA births are associated with many maternal complications such as Cesarean section, postpartum hemorrhage and prolonged hospital stay, and neonatal complications such as shoulder dystocia, neonatal hypoglycemia and increased risks for childhood and adult obesity and diabetes [1]. Antenatal ultrasound is used to diagnose a LGA fetus, which has an estimated weight above the 90^*th*^ centile. One of the primary concerns with LGA fetuses is that they have an increased risk of shoulder dystocia during vaginal delivery, which is when one or both fetal shoulders get trapped in the maternal pelvis during passage through the birth canal. Shoulder dystocia can result in significant fetal and maternal birth related injuries so it is very important that timely diagnosis and monitoring of LGA fetuses occurs so that adverse outcomes can be prevented.

Increased rates of LGA births have been observed in many countries since the late 20^*th*^ century [2–6], in part due to a general rise in maternal body mass index (BMI) [3, 5]. Raised maternal BMI increases rates of gestational diabetes mellitus (GDM), which is a transient state of relative insulin resistance during pregnancy that increases LGA risk. Maternal BMI is derived from height and body weight, and can be used to classify women into four categories: underweight (<18.5 kg/m^2^), normal weight (18.5-24.9 kg/m^2^), overweight (25-29.9 kg/m^2^) and obese (≥30 kg/m^2^). Women who are overweight and obese are at higher risk of LGA than those with normal BMI [7]. The ability to predict LGA births in these high-risk women at an early stage of pregnancy would enable clinicians to apply intervention strategies that prevent the maternal and neonatal morbidity associated with LGA birth. Approaches including adopting a low glycaemic index diet, regular exercise and prevention or treatment of GDM [8] could potentially decrease the incidence of LGA and thus improved maternal and neonatal health outcomes.

Machine learning is a powerful data-driven technique expected to improve prognosis dramatically [9]. However, research on the development of LGA prediction models using machine learning techniques is sparse in the literature. We comprehensively reviewed research articles written in English that were published between January 2015 and June 2022 on PubMed, Embase, ACM and IEEE (accessed on 25 July 2022). The search term used is: (“large-for-gestational-age” OR “large for gestational age”) AND “machine learning”. Six articles were deemed relevant based on their content. Among them, several published models predicted LGA close to delivery, because they included features that are only available close to childbirth: sonographic measurements from 37 weeks gestation [10], gestational weight gain [11], or weight change up to 38 weeks gestation [12]. Therefore, they do not allow interventions to prevent the outcome. Akhtar et al. [13, 14] developed LGA prediction models using data collected from the Chinese National Pre-Pregnancy Examination Program, however, they did not provide sufficient detail on the descriptive features so we are unable to identify the pregnancy stage at which the LGA prediction can be made. Models by Kuhle et al. [15] predicted small and large for gestational age fetus both pre-pregnancy and at 26 weeks gestation. However, all of these published models aim to predict LGA in general pregnancy. There is a lack of studies that focus on a high-risk group, in which LGA risk assessment is more difficult and clinically helpful. Moreover, due to algorithmic complexity, machine learning models often lack explainability, i.e. the ability to provide explanations to clinicians and other users for how model predictions are made. Explainability is a critical component for clinical decision support systems (CDSS) to be adopted effectively in practice [16], and it has been researched in the development of CDSS prototypes [17–21]. Some argue that the lack of explainability is unacceptable in CDSS [22]. Explainability can help users gain insights into the problem; identify potential biases in the models; and generate understanding of why errors occur thereby enabling trust in the models [23, 24]. Additionally, the General Data Protection Regulation (GDPR) in the EU gives data subject “the right not to be subject to a decision based solely on automated processing”, highlighting the need for explainability. None of the previous research on LGA risk prediction explicitly took explainability into consideration during the modeling process.

We have developed models capable of predicting the probability of an LGA birth in women with overweight and obesity at approximately 21 weeks gestation. This was achieved using a combination of maternal characteristics, serum biomarkers and ultrasound findings, in tandem with four machine learning algorithms: random forest, support vector machine (SVM), adaptive boosting (AdaBoost) and extreme gradient boosting (XGBoost). We applied Local Interpretable Model-agnostic Explanations (LIME) [25] to improve the explainability of the models. Our models were designed to be suitable for further development into an easy-to-use CDSS to support LGA screening and the development of targeted intervention strategies. Our findings, which are a progression of the previous work [26], are presented in this paper.

## Materials and methods

**Fig 1:**
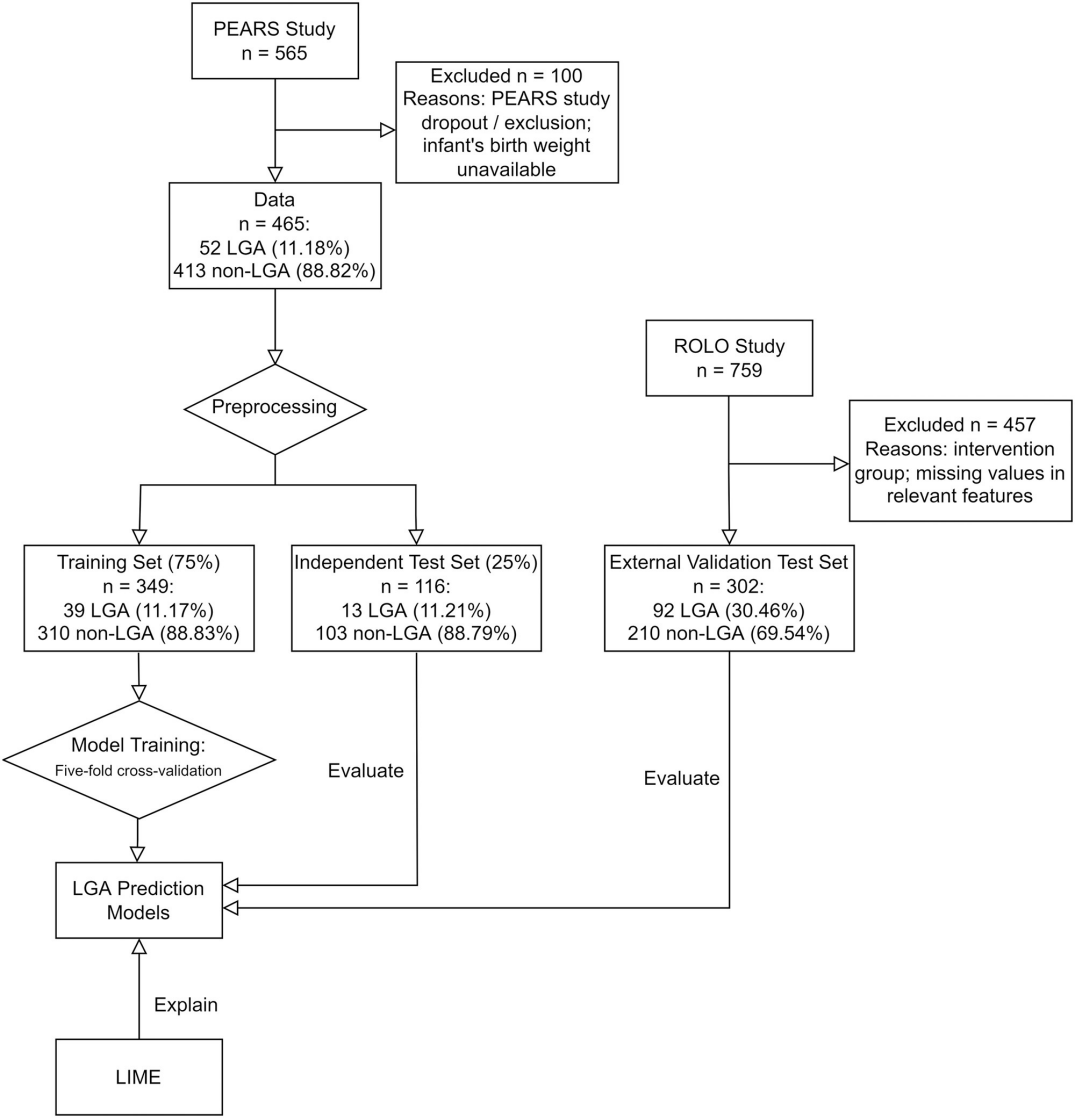
Workflow overview.

Fig 1 shows the overview of the workflow of data preprocessing, model training, evaluation and explanation which are described in detail below.

### Ethics statement

This research is a secondary analysis of the Pregnancy Exercise and Nutrition with smart phone application support (PEARS) study (ISRCTN29316280) and the ROLO (Randomised cOntrol trial of LOw glycaemic index diet vs no dietary intervention in pregnancy to prevent recurrence of a large baby) study (ISRCTN54392969). These randomised controlled trials were carried out at the National Maternity Hospital (NMH), Dublin, Ireland. Ethical approval was granted by the National Maternity Hospital Ethics Committee and maternal written consent was obtained.

### Data

This research used data collected in the PEARS study, a randomised controlled trial of the impact of a multifaceted antenatal lifestyle intervention of low glycaemic index diet, exercise prescription supported with a specifically designed smartphone application, on the incidence of GDM in pregnant women with overweight and obesity (BMI ≥ 25 kg/m^2^) [27]. 565 women with a singleton pregnancy, BMI 25-39.9 kg/m^2^, aged 18 to 45, and that owned a smartphone, were recruited between 10-15 weeks gestation at the NMH between March 2013 and February 2016. Participants were stratified by their BMI, and allocated to either a control group (standard antenatal care) or an intervention group (additional education for lowering glycemic index and setting goals for physical activity). Our investigation involved 465 PEARS study participants that completed the trial and whose babies’ birth weight centiles were available.

We used descriptive features based on clinical data collected during two PEARS study visits, the randomisation or “baseline” visit (14.89±1.65 weeks gestation), and the visit for fetal anatomy scan (21.24±0.89 weeks gestation). At baseline, maternal anthropometry was measured and maternal demographic characteristics were recorded. Maternal blood samples were taken and analysed for several serum biomarkers. At the fetal anatomy scan, fetal biometry was assessed ultrasonographically using a Voluson 730 Expert (GE Medical Systems, Germany) and the estimated fetal weight (EFW) was calculated using the Hadlock formula [28]. Fetal biometry centiles and EFW centiles were also obtained. These features are shown in Table 1.

**Table 1:** Maternal characteristics and serum biomarkers at baseline, and ultrasound findings at fetal anatomy scan at approximately 21 weeks gestation.

Feature	Total (n = 465)	LGA (n = 52)	Non-LGA (n = 413)
**BASELINE**			
Gestational age (weeks)	14.89 (1.65)	14.78 (2.14)	14.90 (1.57)
Maternal age (years)	32.52 (4.38)	32.72 (3.38)	32.50 (4.48)
Ethnicity*			
White Irish	349 (75.05%)	38 (73.08%)	311 (75.30%)
Other White	72 (15.48%)	9 (17.31%)	63 (15.25%)
Black	5 (1.08%)	0 (0%)	5 (1.21%)
Chinese	5 (1.08%)	1 (1.92%)	4 (0.97%)
Other Asian	15 (3.23%)	2 (3.85%)	13 (3.15%)
Mixed	7 (1.51%)	0 (0%)	7 (1.69%)
Not Specified	12 (2.58%)	2 (3.85%)	10 (2.42%)
Education attainment*			
Complete third degree education	288 (61.94%)	36 (69.23%)	252 (61.02%)
Some third degree education	100 (21.51%)	11 (21.15%)	89 (21.55%)
Complete secondary education	54 (11.61%)	1 (1.92%)	53 (12.83%)
Some secondary education	11 (2.37%)	2 (3.85%)	9 (2.18%)
Primary school education	0 (0%)	0 (0%)	0 (0%)
No schooling	0 (0%)	0 (0%)	0 (0%)
Missing	12 (2.58%)	2 (3.85%)	10 (2.42%)
Pobal HP deprivation index	6.10 (11.28)	8.67 (10.58)	5.78 (11.34)
Parity	0.75 (0.92)	0.65 (0.68)	0.76 (0.95)
Height (m)	1.64 (0.07)	1.66 (0.07)	1.64 (0.06)
Weight (kg)	79.22 (10.90)	81.85 (11.87)	78.89 (10.75)
BMI (kg/m^2^)	29.24 (3.32)	29.72 (3.62)	29.17 (3.28)
BMI Category*			
Overweight (25-29.9)	315 (67.74%)	31 (59.62%)	284 (68.77%)
Obese (≥30)	150 (32.26%)	21 (40.38%)	129 (31.23%)
MUAC (cm)	30.98 (2.50)	31.18 (3.26)	30.95 (2.39)
White cell count (10^9^/L)	8.76 (1.88)	8.92 (1.75)	8.74 (1.89)
Fasting glucose (mmol/L)	4.54 (0.34)	4.53 (0.53)	4.55 (0.34)
Insulin (mU/L)	9.32 (4.37)	9.02 (3.93)	9.29 (4.26)
HOMA index	1.85 (0.92)	1.81 (0.86)	1.86 (0.92)
C-peptide (ng/mL)	1.44 (0.67)	1.47 (0.92)	1.43 (0.60)
Total cholesterol (mmol/L)	5.39 (0.94)	5.48 (1.14)	5.38 (0.88)
HDL cholesterol (mmol/L)	1.50 (0.43)	1.46 (0.48)	1.50 (0.41)
LDL cholesterol (mmol/L)	3.25 (0.91)	3.36 (1.03)	3.24 (0.86)
Triglycerides (mmol/L)	1.40 (0.49)	1.46 (0.51)	1.38 (0.47)
C3 (mg/dL)	157.72 (26.60)	157.15 (31.70)	157.79 (26.02)
CRP (mg/L)	3.57 (9.31)	1.67 (1.87)	3.79 (9.79)
Leptin (ng/mL)	42.77 (19.69)	41.56 (20.47)	42.91 (19.65)
Adiponectin (*μ*g/mL)	16.69 (9.57)	16.85 (11.17)	16.67 (9.42)
**FETAL ANATOMY SCAN**			
Gestational age (days)	148.68 (6.25)	148.81 (7.01)	148.66 (6.16)
BPD (mm)	50.18 (3.49)	50.75 (3.44)	50.11 (3.49)
BPD centile (%)	45.70 (24.30)	51.83 (23.90)	44.91 (24.26)
HC (mm)	189.55 (11.69)	192.20 (11.76)	189.20 (11.65)
HC centile (%)	50.95 (21.44)	59.94 (21.04)	49.78 (21.24)
AC (mm)	162.74 (12.38)	166.30 (13.71)	162.27 (12.14)
AC centile (%)	59.70 (23.67)	70.27 (19.25)	58.32 (23.87)
FL (mm)	35.90 (2.86)	36.30 (2.97)	35.84 (2.84)
FL centile (%)	55.31 (22.77)	59.81 (24.41)	54.73 (22.52)
EFW (g)	422.63 (79.44)	441.37 (99.64)	420.20 (76.24)
EFW centile (%)	50.05 (21.24)	59.46 (20.08)	48.83 (21.11)

Values reported are mean (standard deviation) or number (percentage) denoted by *. Body mass index (BMI), mid upper arm circumference (MUAC), homeostatic model assessment (HOMA), low-density lipoprotein (LDL) cholesterol, high-density lipoprotein (HDL) cholesterol, complement component 3 (C3), C-reactive protein (CRP), fetal biparietal diameter (BPD), head circumference (HC), abdominal circumference (AC), femur length (FL) and estimated fetal weight (EFW).

Missing data ranged from 0-64.30% among features, so a threshold of 30% was set for feature inclusion. Features with > 30% missing data were excluded from analysis, resulting in the loss of the serum biomarkers complement component 3 (C3), C-reactive protein (CRP), leptin and adiponectin collected at baseline. Missing data in the included features were imputed using the median for numerical features and mode for categorical features. The dataset was randomly split into a training set of 349 (75%) participants and an independent test set of 116 (25%).

The target feature for our investigation was whether the participant delivered a LGA infant or not. In the PEARS study, infant birth weight centiles were derived from birth weight recorded at delivery corrected for maternal weight, height, parity, ethnicity, final gestational age and infant’s sex, using the Gestation Network’s Bulk Calculator version 6.2.3 UK. If an infant’s birth weight centile was > 90^*th*^, they were deemed LGA.

#### External validation test set

To validate the performance of our models externally, we used data collected from the ROLO study [29]. The ROLO study recruited secundigravid women at the NMH between 2007 and 2011 who had previously delivered a infant weighing greater than 4 kg, and randomised them into the control group (standard antenatal care) or the intervention group (low glycaemic index diet intervention).

A total of 759 women completed the study, and their data were collected, including maternal anthropometry, maternal demographic characteristics and blood biomarkers at baseline (12.95±2.31 weeks gestation), fetal biometry at the fetal anatomy scan visit (20.71±1.25 weeks gestation) measured using a Voluson 730 Expert (GE Medical Systems, Germany), and fetal biometry centiles. At delivery, infant’s birth weight was recorded and Gestation Network’s Bulk Calculator version 6.2.3 UK was used to calculate birth weight centiles corrected for maternal weight, height, parity, ethnicity, gestational age at delivery, and infant’s sex. An infant with birth weight centile > 90^*th*^ was deemed LGA.

Participants in the control group in the ROLO study without missing values in the relevant features were used as an external validation test set.

### Model training and evaluation

In this research, R packages “DMwR” [30], “randomForest” [31], “kernlab” [32], “fastAdaboost” [33], “xgboost” [34], “caret” [35] and “lime” [36] were used for data analysis and modeling in R 3.6.3.

**Fig 2:**
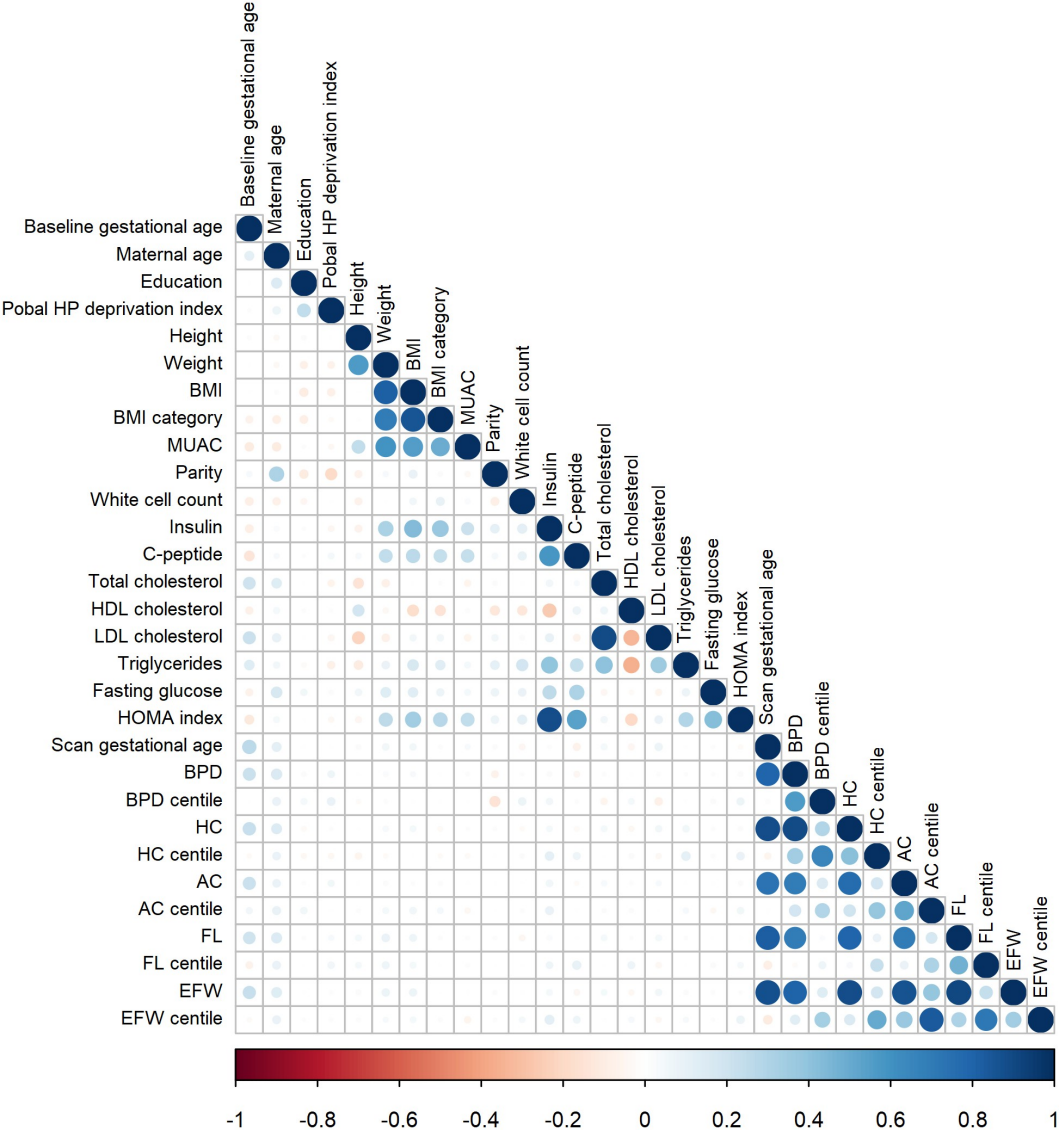
Correlation plot for all numerical and ordinal features. The size of the circles is proportional to the strength of the correlation and the colour represents the direction i.e. a large dark blue circle represents a strong positive correlation and a large dark red circle represents a strong negative correlation. Body mass index (BMI), mid upper arm circumference (MUAC), homeostatic model assessment (HOMA), low-density lipoprotein (LDL) cholesterol, high-density lipoprotein (HDL) cholesterol, fetal biparietal diameter (BPD), head circumference (HC), abdominal circumference (AC), femur length (FL) and estimated fetal weight (EFW).

Some features included in this research are redundant, that is, they carry very similar information as each other. We defined redundant features as those with absolute value of Pearson correlation coefficient greater than 0.6. Fig 2 shows the Pearson correlation coefficient between numerical and ordinal features in the training set. The size of the circles is proportional to the strength of the correlation and the colour represents the direction i.e. a large dark blue circle represents a strong positive correlation and a large dark red circle represents a strong negative correlation. Many highly correlated features were identified. They are: maternal weight, BMI and BMI category; fasting insulin and homeostatic model assessment (HOMA) index; total cholesterol and low-density lipoprotein (LDL) cholesterol; gestational age at fetal anatomy scan, fetal biometry (biparietal diameter (BPD), head circumference (HC), abdominal circumference (AC), femur length (FL)) and estimated fetal weight (EFW); BPD centile and HC centile; AC centile and EFW centile; and FL centile and EFW centile.

**Fig 3:**
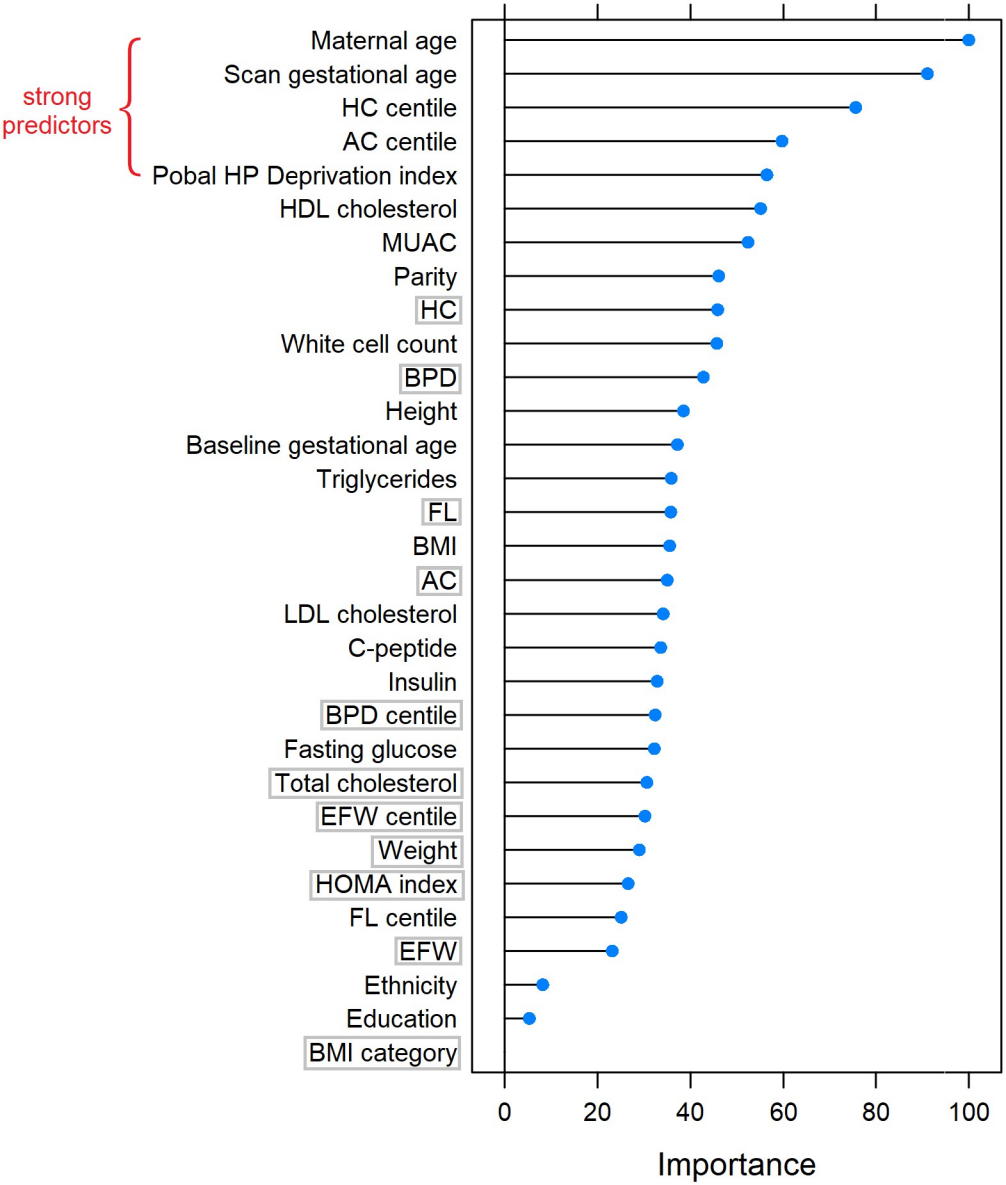
List of features ranked by variable importance for Model 1. Strong predictors are marked in red. Redundant features discarded are marked with grey rectangles. Body mass index (BMI), mid upper arm circumference (MUAC), homeostatic model assessment (HOMA), low-density lipoprotein (LDL) cholesterol, high-density lipoprotein (HDL) cholesterol, fetal biparietal diameter (BPD), head circumference (HC), abdominal circumference (AC), femur length (FL) and estimated fetal weight (EFW).

Two models were developed with different combinations of features for different settings. Model 1 is a feature-agnostic model in which all features were considered candidates, and is suitable for white Irish residents. We ranked the candidate features according to their variable importance based on the mean decrease in Gini coefficient, which measures the features’ contribution to node impurity. Fig 3 shows the ranking of features for Model 1. Maternal age at baseline was ranked highest indicating it is a strong predictor of LGA, followed by gestational age at fetal anatomy scan, HC centile. AC centile and Pobal HP deprivation index (level of affluence and deprivation) were ranked at the top of the list as well. To avoid feature redundancy issue and to reduce the number of inputs required to use the models, which would increase the ease of implementation in a clinical setting, we only included the features with higher variable importance and removed those with less predictive power. As a result, maternal weight, BMI category, total cholesterol, HOMA index, BPD, BPD centile, HC, AC, FL, EFW, and EFW centile (marked with grey rectangles in Fig 3) were removed for Model 1.

Fasting serum biomarkers are not normally collected during routine antenatal visits in Ireland. Their inclusion in a model can have a negative impact on the practicality of adopting the model in a clinical setting. In addition, some features are population-specific. Fetal biometry centiles, EFW centile and BMI category are based on white population. Pobal HP deprivation index, a measurement of the participants’ socioeconomic status based on the geographical area of their residence, is only available to Irish residents. These features will limit the application of the model to other populations. Therefore, we developed Model 2 in which all population-specific features as well as fasting serum biomarkers were excluded. Model 2 is designed to be applicable to pregnant women of all ethnicity and regions in a clinical setting.

**Fig 4:**
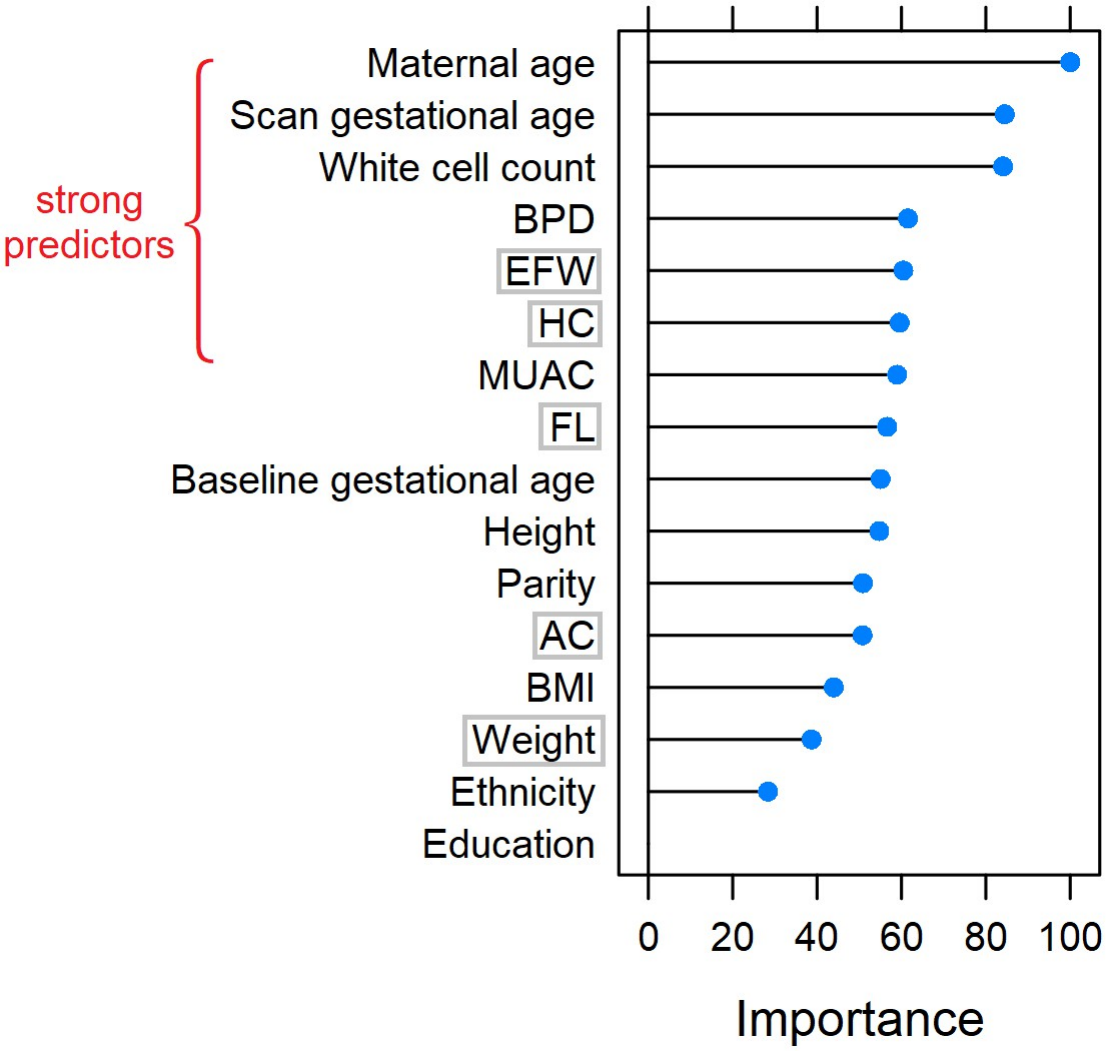
List of features ranked by variable importance for Model 2. Strong predictors are marked in red. Redundant features discarded are marked with grey rectangles. Body mass index (BMI), mid upper arm circumference (MUAC), fetal biparietal diameter (BPD), head circumference (HC), abdominal circumference (AC), femur length (FL) and estimated fetal weight (EFW).

Similar to Model 1, we ranked the candidate features for Model 2, as shown in Fig 4. Maternal age is the most predictive variable, followed by gestational age at fetal anatomy scan and white cell count. BPD, EFW and HC are also important, as well as baseline maternal MUAC. In this model, we allowed gestational age at fetal anatomy scan and fetal biometry to co-exist, because even though they are highly correlated, they carry different medical information and provide implications on fetal biometry centile to support LGA prediction. As a result, the redundant features are baseline maternal weight, HC, AC, FL, and EFW from fetal anatomy scan (marked with grey rectangles in Fig 4), and they were excluded for Model 2.

**Fig 5:**
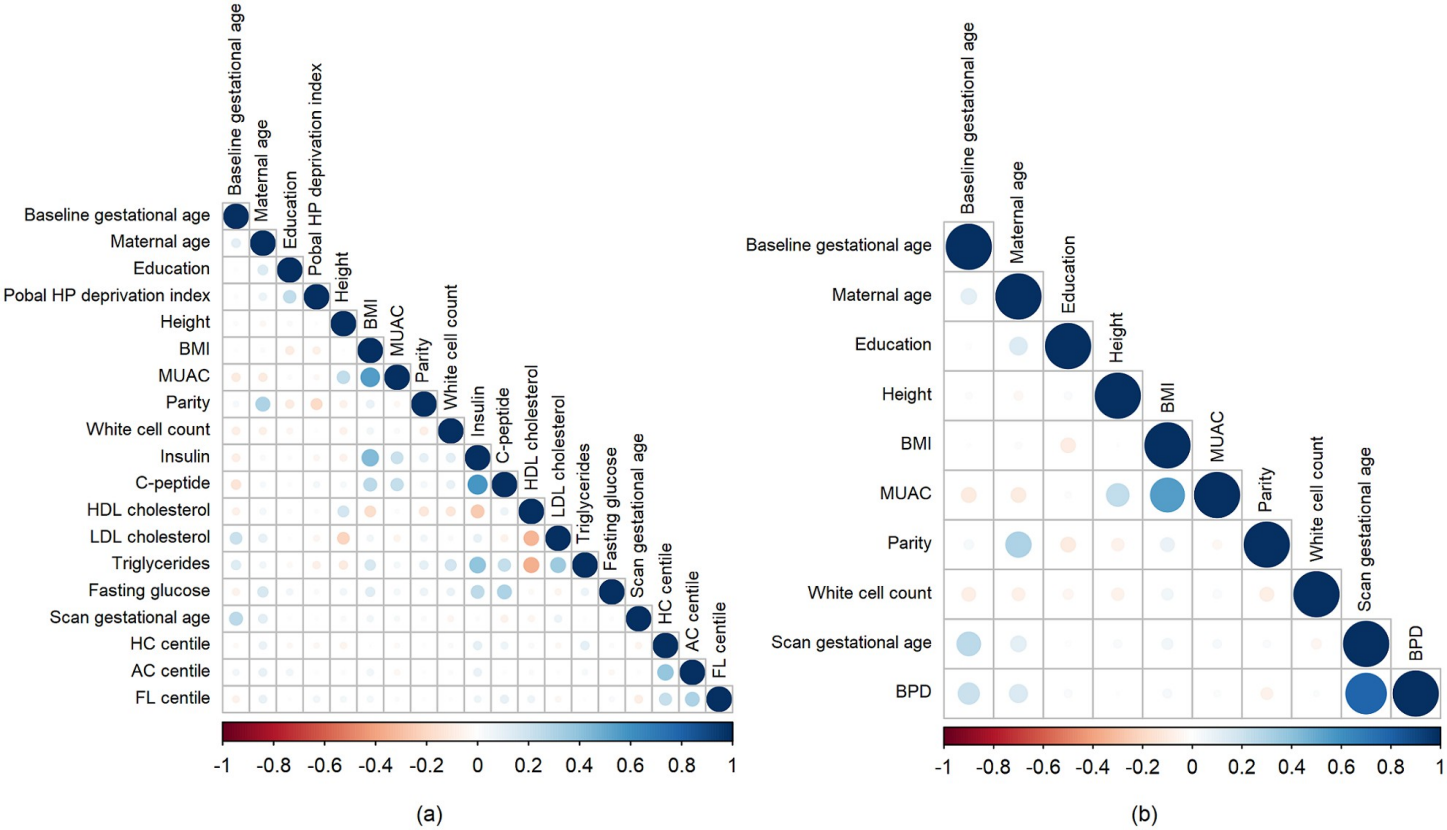
Correlation Plot for Candidate Numerical and Ordinal Features for (a) Model 1 and (b) Model 2. The size of the circles is proportional to the strength of the correlation and the colour represents the direction i.e. a large dark blue circle represents a strong positive correlation and a large dark red circle represents a strong negative correlation. Body mass index (BMI), mid upper arm circumference (MUAC), low-density lipoprotein (LDL) cholesterol, high-density lipoprotein (HDL) cholesterol, fetal biparietal diameter (BPD), head circumference (HC), abdominal circumference (AC) and femur length (FL).

Fig 5 shows the correlation for candidate numerical and ordinal features for Model 1 and 2 after discarding the redundant features. None are highly correlated, i.e. have absolute value of Pearson correlation coefficient > 0.6, with the exception of the gestational age at fetal anatomy scan and BPD in Model 2 which we allowed.

For both Model 1 and 2, Synthetic Minority Over-sampling Technique (SMOTE) [37] was used to balance the training data, and random forest, SVM with linear, polynomial and radial basis function kernels, AdaBoost and XGBoost were applied to develop models to predict class probability. The following parameters were tuned based on the largest area under the receiver operating characteristic curve (AUC-ROC) in five-fold cross-validation: variables randomly sampled as candidates at each split in random forests; polynomial degree (for polynomial kernel), scale (for polynomial kernel) and cost (for both polynomial and radial basis function kernel) in SVMs; the number of trees and method in AdaBoost; the number of boosting iterations, L1 and L2 regularization in XGBoost. Linear booster for XGBoost was used. Other parameters used were set to the default. In cross-validation, only the training folds were balanced by SMOTE and the test folds were unchanged to avoid biased results.

To select the optimal feature subsets, the model fitting process was repeated on the subsets of highly ranked features with different sizes, and the models giving the highest AUC-ROC in cross-validation were selected as the final models.

The models were evaluated on the independent test set using AUC-ROC. Sensitivity, specificity and balanced accuracy (ACC) were calculated at an optimal decision threshold closest to the top-left corner of the ROC curve. The evaluation metrics are calculated as follows:

$$\begin{array}{c} Sensitivity=\frac{TP}{TP+FN} \end{array}$$ (1)

$$\begin{array}{c} Specificity=\frac{TN}{TN+FP} \end{array}$$ (2)

$$\begin{array}{c} Balanced\;ACC=\frac{Specificity+Sensitivity}{2} \end{array}$$ (3)

where:

- True positives (TP): the number of LGA births that are predicted LGA
- False positives (FP): the number of non-LGA births that are predicted as LGA
- True negatives (TN): the number of non-LGA births that are predicted as non-LGA
- False negatives (FN): the number of LGA births that are predicted as non-LGA

Models were also evaluated on the external validation test set to validate our models externally.

### Explainability

Random forest, SVM, AdaBoost and XGBoost are algorithmic complex, and they create “black-box” models that do not reveal any information on its internal logic. To solve this problem and enhance clinicians’ understanding and trust of our models, we improved the local explainability of our models using LIME [25], a well-recognised model-agnostic post hoc interpretation method. LIME learns a local interpretable models around a prediction to explain the predictions of any model in a faithful way. We used LIME to calculate the contributions of descriptive features to a particular prediction to provide insights into how this prediction was made. Then the explanations generated by LIME were validated by case studies.

## Results

The majority of the PEARS participants included were white (90.53%), predominantly white Irish (75.05%). All participants had a least some secondary education and most of them (83.45%) had at least some third degree education. At baseline, maternal age was 32.52±4.38 years, height 1.64±0.07 m, body weight 79.22±10.90 kg and BMI 29.24±3.32 kg/m^2^. 67.74% of the participants were overweight and 32.26% were obese. At delivery, 52 (11.18%) had a LGA birth and 413 (88.82%) delivered a non-LGA infant.

**Fig 6:**
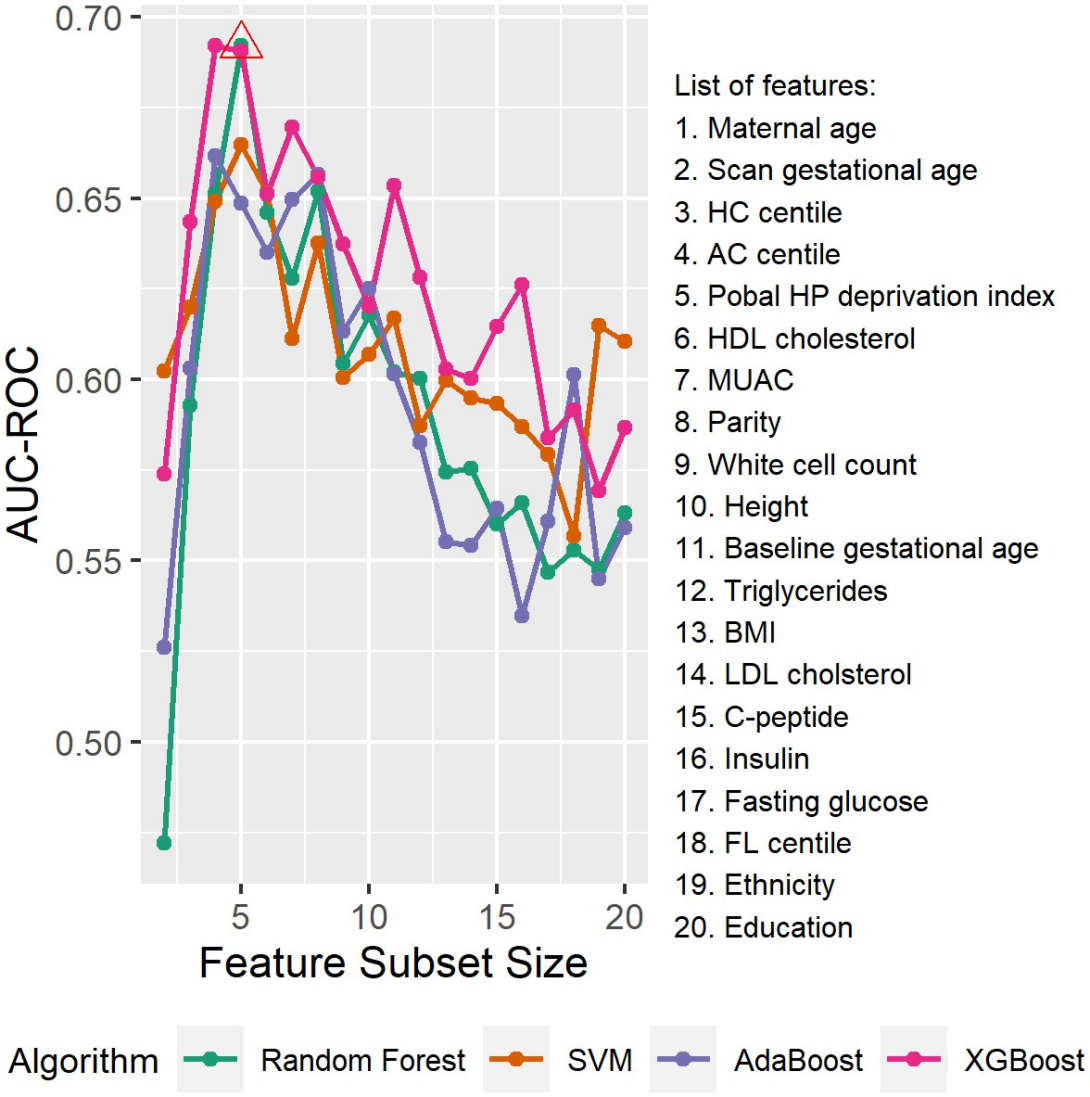
Performance of models using different algorithm and feature subset size for Model 1 in cross-validation. Body mass index (BMI), mid upper arm circumference (MUAC), low-density lipoprotein (LDL) cholesterol, high-density lipoprotein (HDL) cholesterol, fetal head circumference (HC), abdominal circumference (AC), and femur length (FL).

For Model 1, the performance of the models trained using different algorithms and numbers of highly ranked features in cross-validation is compared in Fig 6. AUC-ROC increases overall for all four algorithms when the feature size is small (below 4 or 5), which demonstrates the increase in predictive power as more features were included. As the feature size continues to increase, the performance decreases, possibly due to the “curse of dimensionality”. The highest AUC-ROC in cross-validation is achieved when random forest algorithm is used and the size of the feature subset is five. As a result, we selected random forest as the training algorithm and the top five features as the feature subset for Model 1: maternal age and Pobal HP Deprivation Index at baseline, HC centile and AC centile and gestational age at the fetal anatomy scan.

**Fig 7:**
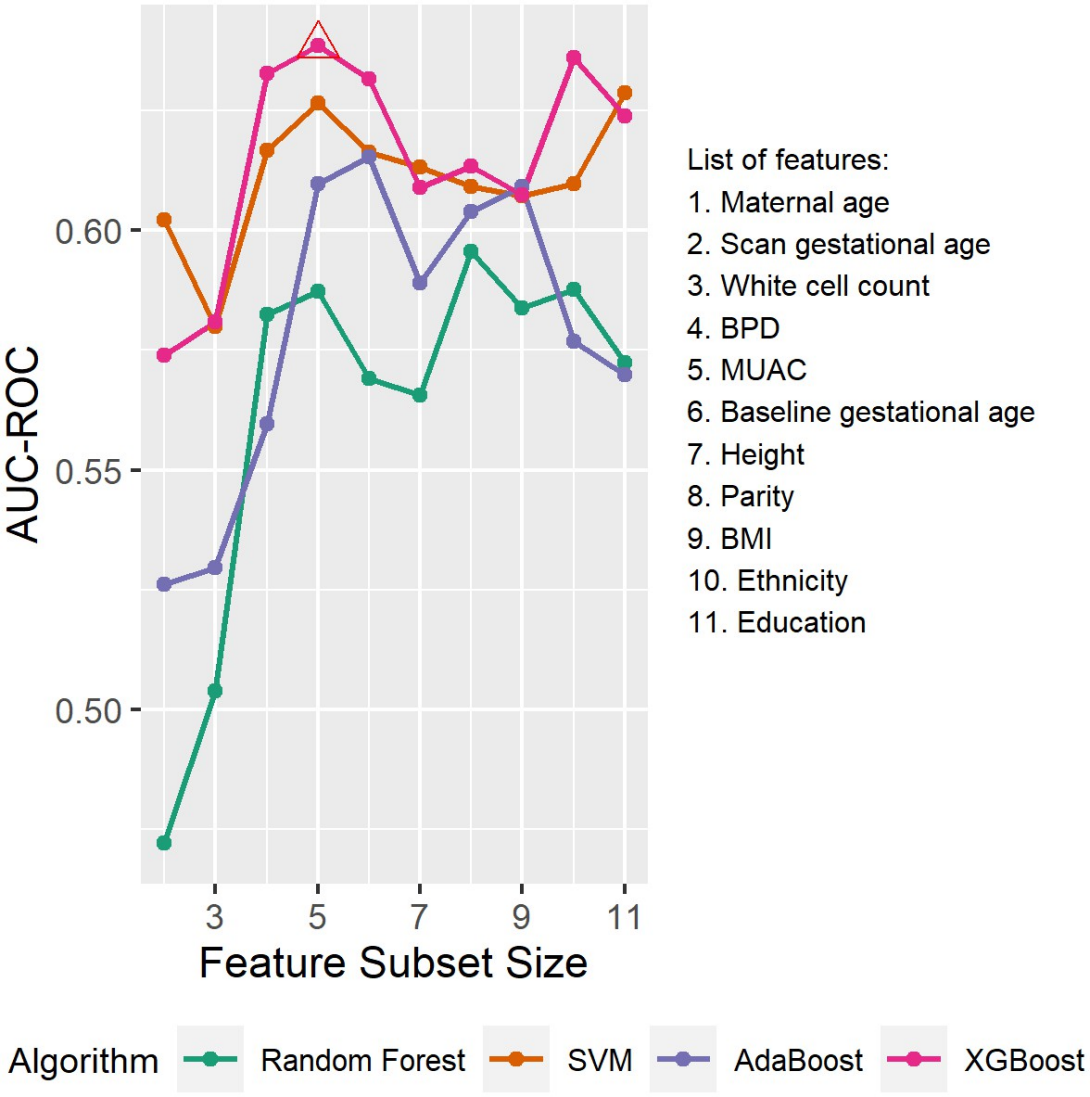
Performance of models using different algorithm and feature subset size for Model 2 in cross-validation. Body mass index (BMI), mid upper arm circumference (MUAC), fetal biparietal diameter (BPD).

Fig 7 compares the performance of the models trained using different algorithms and feature subset sizes for Model 2 in cross-validation. An overall increasing trend in AUC-ROC is observed when the feature subset size is small (below 5), possibly because of the information gained through adding new features. As the number of features further increases, the performance levels off with fluctuations. The highest AUC-ROC in cross-validation is achieved when using XGBoost at feature subset size of five. Therefore, XGBoost was selected and the top five features were included: maternal age, MUAC and white cell count at baseline, and BPD and gestational age at the fetal anatomy scan.

We conclude that maternal age and gestational age at fetal anatomy scan (highly correlated with fetal biometry and EFW) are important features for LGA prediction, as they are ranked high in both models (see Figs 3 and 4). White cell count, BPD and MUAC are also important as they are included in Model 2. Of the population-specific features, Pobal HP deprivation index, HC centile and AC centile are the most important ones.

Table 2 shows the performance of our final models evaluated on the independent test set. Model 1 performs well overall, with an AUC-ROC of 0.75. At a decision threshold 0.36, Model 1 was able to predict 85% of LGA participants and 62% of non-LGA participants correctly, achieving a balanced accuracy of 73%. Six participants in the independent test set are non-white and all of them had a non-LGA birth. 67% of them were predicted as LGA incorrectly, indicating that our model which is suitable for white Irish residents may not generalise well to other populations. Model 2 achieved an AUC-ROC of 0.57. At the optimal decision threshold of 0.34, Model 2 was able to predict 54% of LGA participants and 71% of non-LGA participants correctly, achieving a balanced accuracy of 62%. The performance of Model 2 is lower than that of Model 1, possibly because of the lost of the information contained in the population-specific features, especially Pobal HP deprivation index, HC centile and AC centile which are strong predictors of LGA.

**Table 2:** Performance of Models 1 and 2 on the independent test set.

Model	Algorithm	No. of features	AUC-ROC	Threshold	Sensitivity	Specificity	Balanced accuracy
1	Random forest	5	0.75	0.36	0.85	0.62	0.73
2	XGBoost	5	0.57	0.34	0.54	0.71	0.62

Independent test set: 13 LGA (11.21%), 103 non-LGA (88.79%).

**Fig 8:**
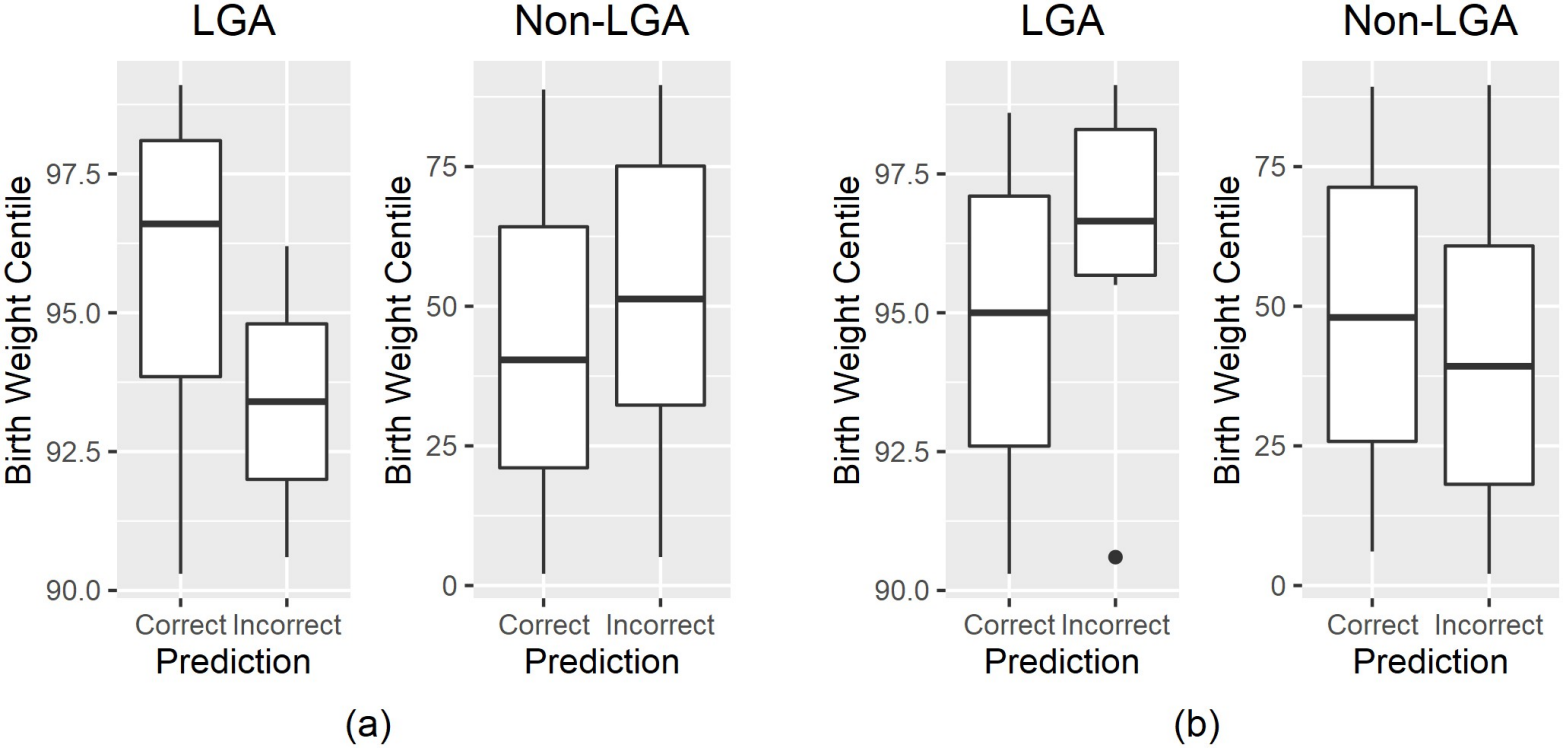
Comparison of Birth Weight Centile Between Correctly and Incorrectly Predicted Independent Test Cases for (a) Model 1 and (b) Model 2. Model 1 is more likely to give an incorrect predictions for cases closer to borderline.

We compared the birth weight centiles of the participants in the independent test set who were predicted correctly and incorrectly by our models, as shown in Fig 8. Of all LGA participants, those incorrectly predicted as non-LGA (false negatives) by Model 1 had overall lower birth weight centiles than those correctly predicted (true positives). Of all non-LGA participants, those incorrectly predicted as LGA (false positives) by Model 1 had overall higher birth weight centiles than those correctly predicted as non-LGA (true negatives). This demonstrates that our Model 1 is more likely to give incorrect predictions for borderline cases, i.e. those whose birth weight centiles are close to the cutoff of the 90^*th*^ centile for defining LGA from non-LGA cases. Model 2 did not show similar patterns, possibly because the model does not demonstrate a very high performance on the independent test set.

### External validation

We validated Model 1 using an external validation test set of 302 ROLO participants from the control group who had no missing values in the features included in Model 1. Most of these participants were white (97.02%), predominantly white Irish (88.41%). All participants had at least some secondary education and the majority (67.88%) of them had at least some third degree education. At baseline, maternal age was 32.37±4.34 years, height 165.67±6.20 cm, body weight 73.07±13.08 kg and BMI 26.64±4.64 kg/m^2^. 0.66% of these participants were underweight, 42.38% were at a normal weight, 37.75% were overweight and 19.21% were obese. At delivery, 92 (30.46%) had an LGA birth and 210 (69.54%) delivered a non-LGA infant.

Model 1 achieved acceptable performance on this external validation test set, giving an AUC-ROC of 0.62. This is not as good as the performance on the independent test set from the PEARS dataset, potential because of the difference between PEARS and ROLO populations. PEARS participants were all women with overweight and obesity, whereas some ROLO participants were underweight or at a normal weight but all had a macrosomic birth history. The rate of LGA is different (11.18% in PEARS versus 30.46% in the external validation test set from ROLO). Unfortunately, we are unable validate Model 2, because white cell count was not collected in the ROLO study.

### Explainability and case study

**Fig 9:**
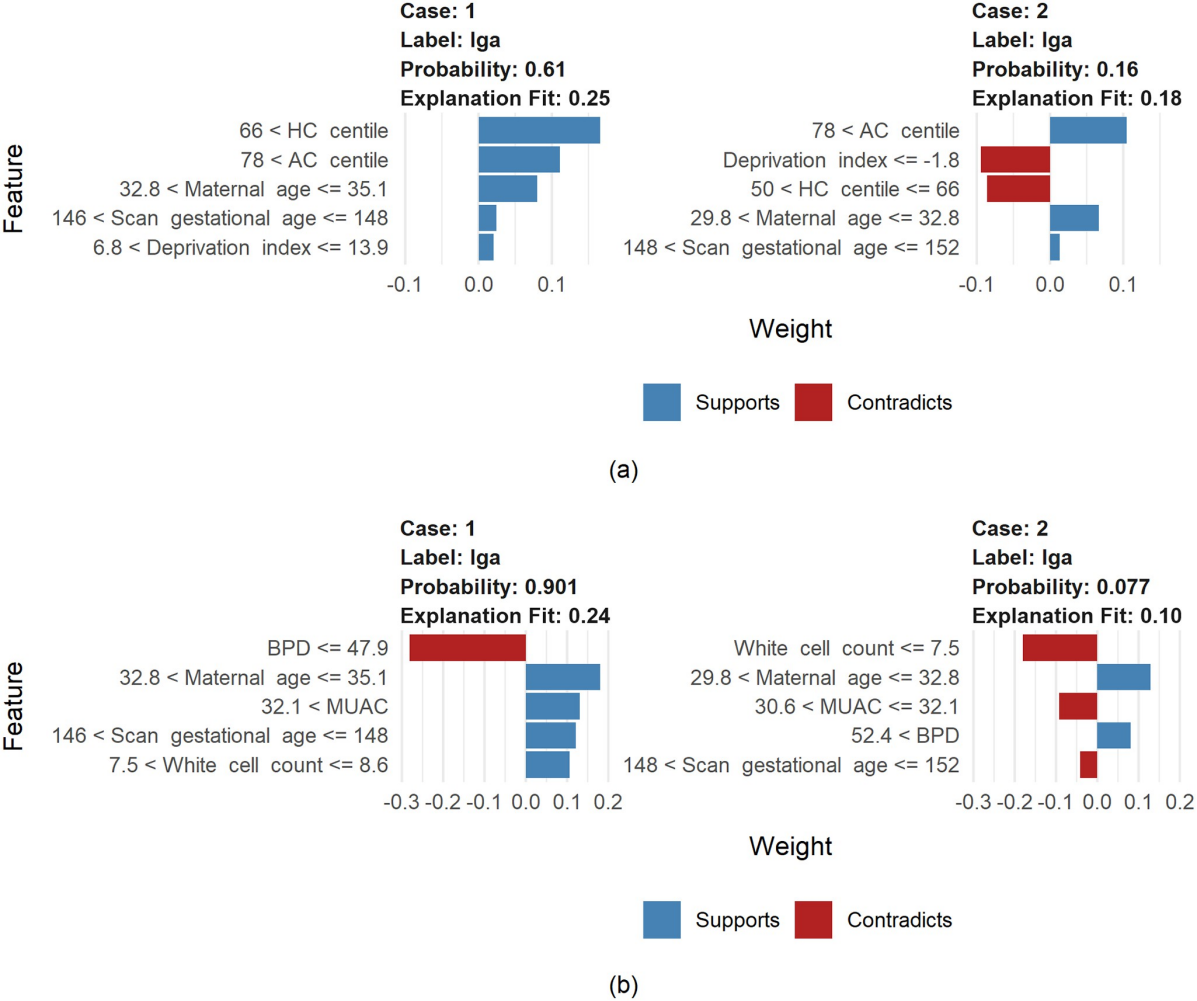
Feature Effects on the LGA Class and Predicted LGA Probability for Case 1 (LGA) and Case 2 (non-LGA) in (a) Model 1 and (b) Model 2 Using LIME. Case 1 has extremely high MUAC and HC centile and extremely low BPD. Case 2 has extremely high BPD and extremely low Pobal HP deprivation index and white cell count. Both cases have extremely high AC centile. Mid upper arm circumference (MUAC), fetal biparietal diameter (BPD), head circumference (HC), abdominal circumference (AC).

LIME was applied to explain the models locally. Two cases from the independent test set were used for case studies: an LGA case, and a non-LGA case. They are shown in Table 3. We define extreme values of a feature as values greater than or equal to the third quartile or lower than the first quartile of the feature prior to imputation, and a normal range as values between the first and third quartile. Both cases have extremely high AC centile. Case 1, the LGA case, has extremely high maternal MUAC and HC centile and extremely low BPD. Case 2, the non-LGA case, has extremely high BPD and extremely low Pobal HP deprivation index and white cell count. Fig 9 shows the lists of features ranked by their effects on the LGA class using LIME as well as the predicted class probability for LGA. Both cases were predicted correctly by our models, indicating that the models perform well on these cases and these cases are valid for cases studies. We aim to investigate how these extreme values affect the predicted risk, and which features have opposite effects on Case 1 and 2 to explain the prediction difference.

**Table 3:** Test cases.

Case	Class	Maternal age	MUAC (cm)	Pobal HP deprivation index	White cell count (10^9^/L)	Scan GA (days)	BPD (mm)	HC centile (%)	AC centile (%)
1	LGA	33.35	**35.9**	11.4	8.2	147	**45.4**	**67**	**82**
2	non-LGA	32.33	31.9	**-16**	**6.7**	151	**53**	66	**86**

Case 1 has extremely high maternal MUAC and HC centile and extremely low BPD. Case 2 has extremely high BPD and extremely low Pobal HP deprivation index and white cell count. Both cases have extremely high AC centile. Mid upper arm circumference (MUAC), gestational age (GA), fetal biparietal diameter (BPD), head circumference (HC), abdominal circumference (AC).

In Model 1, the extremely high AC centile largely increases the LGA risk for both Case 1 and 2. Case 1’s extremely high HC centile highly increases the LGA risk, whereas Case 2’s HC centile in normal range decreases the risk, leading to the opposite predicted classes for these cases. The extremely low Pobal HP deprivation index in Case 2 decreases the LGA risk greatly, whereas Pobal HP deprivation index in normal range for Case 1 increases the risk, contributing to opposite classes as well.

In Model 2, white cell count and BPD contribute highly to the different predicted classes. The extremely low white cell count for Case 2 decreases the LGA risk to a large extent, while the white cell count in normal range for Case 1 increases the LGA risk. The extremely low BPD leads to a big decrease in the LGA risk for Case 1, whereas the extremely high BPD increases the risk for Case 2. In addition, the extremely high MUAC for Case 1 increases the LGA risk whereas MUAC in normal range for Case 2 decreases the risk. Gestational age at fetal anatomy scan contributes to the difference of predictions as well.

As a result, the feature effects on these predictions are rational, showing that LIME performs well in providing explanations for the predictions of our models. It indicates that LIME has potential for explaining our models locally in a clinical setting.

## Discussion

The following factors were found to be important in the prediction of an LGA infant: maternal age, MUAC, white cell count at baseline, BPD and gestational age at fetal anatomy scan, as well as Pobal HP deprivation index, HC centile and AC centile which are population-specific to white Irish residents.

Applying machine learning algorithms to readily available clinical data may support the early and accurate identification of LGA births during the second trimester of pregnancy. Our Model 1 is suitable for the prediction of LGA births for white Irish residents and achieved high overall performance (AUC-ROC of 0.75). It compares favourably with Kuhle et al. [15], who applied logistic regression, elastic net, classification tree, random forest, gradient boosting and neural network on data collected from mothers resident in Nova Scotia, and achieved AUC-ROC of 0.563-0.594 and 0.659-0.700 in pre-pregnant nulliparous and multiparous women, respectively, and AUC-ROC of 0.673-0.705 and 0.718-0.748 in nulliparous and multiparous women at 26 weeks gestation, respectively. Our findings are applicable to the mid trimester of pregnancy, whereas most published machine learning models were only able to predict LGA in late pregnancy or close to delivery [10–12], or provided insufficient information on the timing of prediction [13, 14]. Our models predict LGA when the fetal size is much smaller, though a tradeoff in reduced performance was observed.

Machine learning models highly depend on the data they are trained on. Based on where participants were recruited, the published models may only be applicable to areas with similar population and antenatal care, while none has evaluated or discussed the applicability and generalisability of their models. The data for this study was collected at a single centre in Dublin, Ireland, where the majority of participants were white. Of PEARS participants included in this study, 75.05% were white Irish, and 15.48% were from other white origins. Only 6.88% of the participants were from other ethnic origins, including Black, Asian and mixed. As most of the participants were white Irish residents, two models suitable for clinical uses were developed: Model 1 for white Irish residents for which our initial cross-population evaluation showed lack of generalisability for non-white women, and Model 2 for pregnant women of all ethnicity and regions. The external validation of Model 1 gave acceptable performance but was less accurate than the performance on the independent test set, potentially because of the difference in populations, especially regarding maternal BMI and macrosomic birth history. Future work on validation on larger datasets and on other populations, such as non-white women, is required before application in clinical settings.

In addition, this is the first LGA study to target women with overweight and obesity, known to be at-risk of having a LGA infant. Lifestyle interventions in pregnancy are cost-effective in reducing pregnancy complications [38] therefore earlier prediction of at risk pregnancies hold significant potential to offer interventions to at risk women. Although we did not find any previous machine learning studies in the prediction of LGA among pregnant women with overweight and/or obesity, we found some studies conducted on GDM prediction among these women. Balani et al. [39] developed a decision tree for obese pregnant women based on body composition analysis. Their model, trained on data from pregnant women in UK of which the majority is Caucasian, may only work in regions with similar population and body composition analysis machine. Du et al. [19] developed SVM models to predict GDM in women with overweight and obesity. Their models, based on the white population, have the potential to generalise to a non-white population, however, a lower decision threshold might be needed. Future work could be conducted to investigate if differing decision thresholds are required for different populations in LGA prediction.

Several CDSSs developed to improve pregnancy care have been tested in clinical settings [40–43]. However, most of them are based on clinical guidelines and knowledge. Only Caballero-Ruiz et al. [42] who evaluated their CDSS in a clinical trial incorporated some machine learning techniques in their system. In contrast, our research focusing on the development of a machine learning-based CDSS, which is data-based and does not directly rely on medical knowledge, could help to bridge the gap in literature.

Clinicians have heavy workloads and time constraints may be a barrier to the use of CDSS [44]. We took clinical practicality into account in many aspects of model development. For instance, we used probabilistic prediction so that our models’ false positive rate would be adjustable to suit the needs in different clinical settings. In the feature selection step, we reduced redundant features to keep the minimum number of features so that the models would be fast to use and thus save clinicians’ time. This enhances the practicality of applying our models in clinical practice and facilitates efficient clinical workflow. Screening a high-risk group instead of the whole population is time-saving as well as cost-effective, and can be facilitated by the use of these models. Model 1 was developed in a feature-agnostic manner, however, for Model 2 we considered the difficulty of data collection as an initial feature selection step in modeling. Therefore, in Model 2, fasting serum biomarkers were excluded at the beginning because they are not routinely assessed in an Irish clinical setting. Moreover, Model 1 excluded fasting serum biomarkers in the final feature set as they were not highly ranked as informative. As a result, the models we developed included clinically available features only without the need to acquire additional fasting blood samples. They are expected to be easily applicable in an antenatal routine and lead to a more widely usable CDSS.

Furthermore, the inclusion of explainability added more novelty to our work. LIME was applied to explain the predictions of our models, and explanations were proven effective by case study. Explainability in CDSSs has been reported to boost the decision confidence for clinicians and increase the acceptability and trustworthiness of these systems [16]. Although there is a debate on whether adding explainability in artificial intelligence-based systems is necessary and worth the substantial cost [23], explainability may enhance the uptake of CDSSs and may become a requirement in the future because of societal, regulatory and ethical pressures [16]. As a result, we believe that the use of LIME increases the potential of using our models in a clinical setting.

Another advantage of our research is the appropriate handling of the class imbalance problem. In medical datasets, it is common that healthy cases outnumbers the pathological cases, often significantly, thus leading to severe class imbalance in the target feature. This is also the case in our research as our primary dataset consisted of 52 (11.18%) LGA cases and 413 (88.82%) non-LGA cases. Without handling this issue correctly, the models developed may perform well on the majority class while under-performing on the minority classes. We balanced the training data using SMOTE, which draws lines connecting similar cases in the minority class and synthetically creates new ones along the lines [37]. Moreover, we used balanced accuracy instead of accuracy when evaluating on the independent test set, because the latter may give a biased evaluation on unbalanced data.

We treated the PEARS study as a cohort study without taking into account the effect of the intervention. Using the entire cohort for analysis was beneficial for this research because it captured a range of fetal gestational weights and doubled the size of the dataset which allows for more accurate modeling.

## Conclusion

We successfully developed LGA prediction models for pregnant women with overweight and obesity at approximately 21 weeks gestation using random forest and XGBoost algorithms, and we explained the predictions of our models by feature effects on these predictions based on LIME. Our explainable model for white women demonstrates good performance with potential to be developed into a CDSS for clinical use to identify in early pregnancy women at risk for LGA infant. Earlier identification in pregnancy allows for the development of appropriate interventions to reduce the maternal and neonatal morbidity associated with the birth of an LGA infant.
